# Review on the Development and Applications of Medicinal Plant Genomes

**DOI:** 10.3389/fpls.2021.791219

**Published:** 2021-12-23

**Authors:** Qi-Qing Cheng, Yue Ouyang, Zi-Yu Tang, Chi-Chou Lao, Yan-Yu Zhang, Chun-Song Cheng, Hua Zhou

**Affiliations:** ^1^State Key Laboratory of Quality Research in Chinese Medicine, Faculty of Chinese Medicine, Macau University of Science and Technology, Taipa, Macao SAR, China; ^2^Lushan Botanical Garden, Chinese Academy of Sciences, Jiujiang, China; ^3^Joint Laboratory for Translational Cancer Research of Chinese Medicine, The Ministry of Education of the People’s Republic of China, Macau University of Science and Technology, Taipa, Macao SAR, China

**Keywords:** medicinal plant, genome, sequencing, long-read sequencing technology, application

## Abstract

With the development of sequencing technology, the research on medicinal plants is no longer limited to the aspects of chemistry, pharmacology, and pharmacodynamics, but reveals them from the genetic level. As the price of next-generation sequencing technology becomes affordable, and the long-read sequencing technology is established, the medicinal plant genomes with large sizes have been sequenced and assembled more easily. Although the review of plant genomes has been reported several times, there is no review giving a systematic and comprehensive introduction about the development and application of medicinal plant genomes that have been reported until now. Here, we provide a historical perspective on the current situation of genomes in medicinal plant biology, highlight the use of the rapidly developing sequencing technologies, and conduct a comprehensive summary on how the genomes apply to solve the practical problems in medicinal plants, like genomics-assisted herb breeding, evolution history revelation, herbal synthetic biology study, and geoherbal research, which are important for effective utilization, rational use and sustainable protection of medicinal plants.

## Introduction

Medicinal plants, in the simple definition, are plants that can be used for medicinal purposes; in the detailed definition, are plants that have been verified and used for a long time as traditional medicines, have been found to have medicinal value in modern research, or contain medicinal ingredients in them. And they can provide the essential resources for human life, such as drugs, nourishment, condiments, and medicinal oil. They also uncovered and promoted the evolution of nature, animals, and humans. The foundation of all life is the genetic code. Therefore, access to the primary DNA sequence and how genes are encoded within the genome has become a basic resource in biology (Hamilton and Robin Buell, 2012). The genomics study of medicinal plants is to elucidate their molecular mechanism to prevent human diseases, by utilizing the genetic information and regulatory network of the species and the omics technologies, accordingly, to reveal their effect on the human body from the level of the genome. Now the process of genome sequencing in plants lags behind that in microorganisms and animals. Due to the lack of genomic information, there is a lack of communication between medicinal plants and modern life sciences, and the new frontier life science technology is hardly be applied to their research. Over the years, the works of research on medicinal plant medicines mainly focus on chemistry and pharmacology, the studies to uncover the biological nature of medicinal plants need to be strengthened.

Regarding plant genome sequencing methods and strategies, radical changes have taken place in the past 5 years, and medicinal plant genome sequencing is no exception. Previous reviews summarized the status of sequenced plant genomes before 2012 (Hamilton and Robin Buell, 2012), the status of sequenced angiosperm genomes before 2018 (Chen et al., 2018), and the impact of third generation genomic technologies on plant genome assembly (Jiao and Schneeberger, 2017). In addition, there were also Chinese reviews that proposed and introduced the Herb Genome Program (Chen et al., 2010) and 1,000 genome projects of medicinal plants (Chen et al., 2019). As sequencing cost reduces drastically and long-read sequencing technology develops quickly in recent years, it is certain that the genome continues to be improved, while more and more large and complicated medicinal plant genomes are reported. The future of revealing the secret of medicinal plant biology is bright. However, there is still not a review covering the medicinal plant genomes that have been released so far and introducing the development of sequencing strategies and applications.

In this manuscript, we conducted a systematic review of medicinal plants genome research. Moreover, the genome situation, sequencing technology development, and application of medicinal plant genomes are discussed. This review provides a historical perspective on the current situation of genomes in medicinal plant biology and highlights the use of rapidly developing sequencing technologies in plant biology. Challenges in genomics for medicinal plants are improved to some extent by long-read sequencing technologies regarding the current limitations. Multiple omics methods are integrated to make better use of medicinal plant genome data and to solve practical problems meeting in the breeding and medical fields. We also conduct a comprehensive summary of the application of medicinal plant genomes, to promote the studies of important questions in plant biology, like genomics-assisted herb breeding, herbal synthetic biology, and geoherbal research, which are significant for securing the future of medicinal plants and their active compounds.

## Literature Search Methods and Results

**FIGURE 1:**
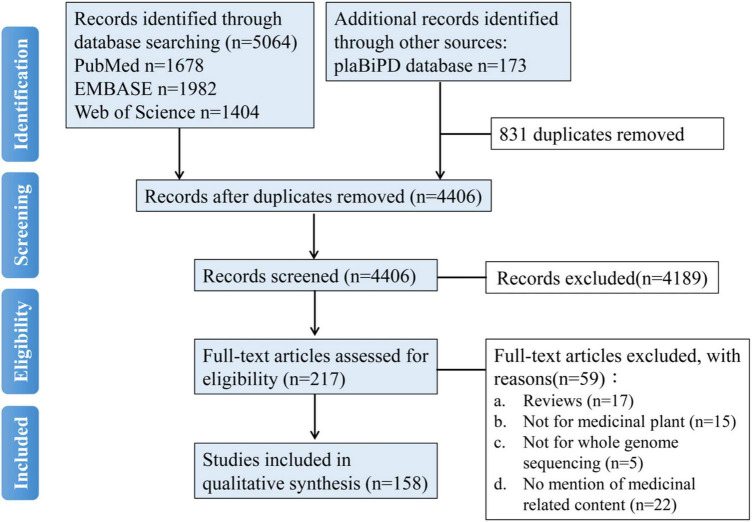
A flowchart of literature search and selection for a systematic review about medicinal plant genomes.

The systematic literature search was performed by the following PRISMA guidelines (Moher et al., 2009). Firstly, it was performed through electronic databases, including PubMed (National Library of Medicine, United States), EMBASE (Elsevier, Netherlands), and Web of Science (Clarivate Analytics, United States) databases published until June 4, 2021. Studies were selected using the term “medicinal plant genome.” Additionally, we also searched the studies from the plaBiPD (Forschungszentrum Jülich GmbH, Germany) database and identified the medicinal plants from all the plants which have reported genomes. About the medicinal plant genomes, a total of 5,064 articles were identified initially by retrieving the electronic database comprehensively. Among these, 1,678 articles were from PubMed, 1,982 articles were from EMBASE, and 1,404 articles were from Web of Science, 173 articles were from the plaBiPD database, 831 articles were excluded for duplicates. A total of 4,189 articles were excluded by scanning the titles and the abstracts, and the exclusion reasons included irrelevant articles, not studies, and so on. Fifty-nine articles were excluded by reading the full-text manuscripts, with the exclusion reasons of reviews, not for medicinal plants and not for whole-genome sequencing and no mention of medicinal related content. Finally, a total of 158 articles were included in this meta-analysis. A flowchart of articles search and selection is shown in Figure 1. According to our statistical result, there were at least 161 reference genomes reported in 158 articles belonging to 126 medicinal plants published. We counted the number of journals that have published medicinal plant genomes, there were a totally of 40 journals, and the corresponding journal name and article number are provided in Supplementary Table 1. Since 2010, articles about medicinal plant genomes have appeared in journals almost every year. Since 2017, the number of medicinal plant genome articles has increased significantly.

## General Introduction of Medicinal Plant Genomes Research

### History and General Characteristics of Medicinal Plant Genome Research

**FIGURE 2:**
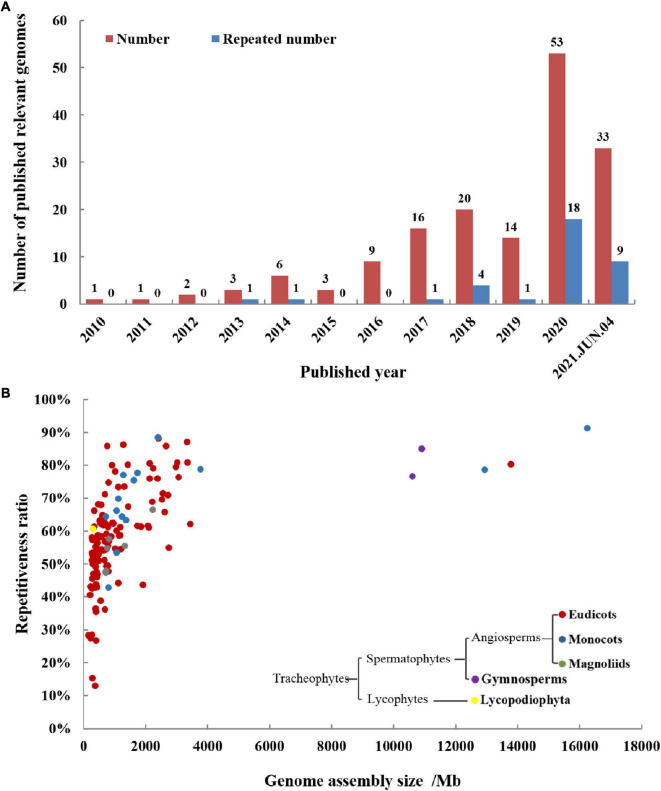
Publication history **(A)** and general information **(B)** of medicinal plant genomes. **(A)** The total number and repeated sequencing number of medicinal plant genomes are increasing year by year, proving that it has received more and more scientific research attention. **(B)** The figure shows published medicinal genome assemblies analyzed for genome-wide repeat levels and genome size. The repetitiveness of most medicinal plant genomes is generally high and correlated to genome size. The sequenced medicinal plants are divided into five groups based on phylogeny, including lycopodiophyta, gymnosperms, eudicots, monocots, and magnoliids, and eudicot accounts for the majority of them.

The medicinal plant genomes are more complex than animal genomes, so the process of sequencing the medicinal plant genomes has been hindered, and it entered a period of rapid development from 2016. This may be due to the decline in sequencing price and the development of long-read sequencing technologies. The status of medicinal plant genomes articles reported each year is shown in Figure 2A. In 2020, the number of published medicinal plant genomes has reached up to 53. In 2021, 33 medicinal plant genome articles have been published until June 4th, and the total article number is inferred to be more than 60. As more and more medicinal plant genomes have been revealed, several plants have been sequenced twice or multiple times for genomes. Among these repeatedly sequenced medicinal plant genomes, some are because of sequencing at the same time, some are due to improved level and quality, and some are genomes of different varieties from the same species. Among those 53 medicinal plant genomes reported in 2020, 18 genomes were reported repeatedly, accounting for 34%. This tells us that sequencing technology is continuously developing and progressing, bringing us to a completer and more accurate genome. Take *Panax notoginseng* (Chinese name: Sanqi) as an example, five versions of its genomes have been reported, the first two versions published in 2017 were sequenced by the next-generation sequencing (NGS) technology of Illumina platform (Chen et al., 2017; Zhang D. et al., 2017), and the recent three versions published in 2020 and 2021 were sequenced by the third-generation sequencing technologies of Pacific Biosciences (PacBio) and Oxford Nanopore (ONT) (Fan G. et al., 2020; Jiang et al., 2021; Yang et al., 2021b). The latest two versions of the genome were assembled to the chromosome level, the length of the assembled sequences was hundreds of times longer than the first two versions, and the accuracy and credibility of annotation have also been greatly improved. The statistical results of detailed information about medicinal plant genomes were shown in Table 1.

**TABLE 1:** The statistical results of medicinal plant genome published journals.

#	Name	Platform	Class	Esti-size	Assem-size	Repeat	Contig N50	Scaffold N50	Gene	References
				Mb	Mb	%	kb	kb	#	
1	*Acer truncatum*	I, P	eudi	739	633	61.8	773.2 kb	46.36 Mb	28438	Ma Q. et al., 2020
2	*Akebia trifoliata*subsp.*australis*	I, P	eudi	670	682	71.2	6.2 Mb	43.11 Mb	25598	Huang et al., 2021
3	*Allium sativum*	I, P, O	mono	16900	16243	91.3	194 kb	1691 Mb	57561	Sun et al., 2020
4	*Aloe vera*	I, O	mono	16040	12930	78.7	NA	14.6 kb	86177	Jaiswal et al., 2021
5–1	*Andrographis paniculata*	I, P	eudi	280	269	53.3	388 kb	NA	25428	Sun et al., 2019
5–2	*Andrographis paniculata*	I, P	eudi	310	284	57.4	5.14 Mb	NA	24015	Liang et al., 2020
6–1	*Apium graveolens*	I	eudi	3180	2210	68.9	13.1 kb	35.6 kb	34277	Li M.-Y. et al., 2020
6–2	*Apium graveolens*	I, P	eudi	3470	3330	87.1	790.6 kb	289.78 Mb	31326	Song et al., 2021
7–1	*Aquilaria sinensis*	I, O	eudi	773	727	59.1	1.1 Mb	88.78 Mb	29203	Ding et al., 2020
7–2	*Aquilaria sinensis*	I	eudi	784	784	61.2	NA	87.6 Mb	35965	Nong et al., 2020
8	*Aquilegia oxysepala* var. *kansuensis*	I, P	eudi	312	293	45.7	2.2 Mb	40.9 Mb	25571	Xie et al., 2020
9	*Artemisia annua*	I, P, R	eudi	1740	1740	61.6	18.95 kb	104.86 kb	63226	Shen et al., 2018
10	*Asparagus setaceus*	I, O	mono	720	710	64.4	1.36 Mb	2.19 Mb	28410	Li S.-F. et al., 2020
11–1	*Averrhoa carambola*	I, O	eudi	358	335	61.3	4.22 Mb	31.25 Mb	25419	Wu S. et al., 2020
11–2	*Averrhoa carambola*	I	eudi	475	471	68.2	44.84 kb	2.76 Mb	24726	Fan Y. et al., 2020
12	*Azadirachta indica*	I	eudi	364	364	13.0	740 bp	452 kb	20169	Krishnan et al., 2012
13	*Betula platyphylla*	I, P	eudi	430	430	43.0	751 kb	NA	31253	Chen S. et al., 2020
14	*Brassica oleracea*	I, R, Sa	eudi	630	540	38.8	26.8 kb	1.46 Mb	45758	Liu S. et al., 2014
15	*Broussonetia papyrifera*	I, P	eudi	380	387	49.2	171.2 kb	29.48 Mb	30512	Peng et al., 2019
16	*Calotropis gigantea*	I	eudi	225	157	28.3	48.6 kb	806.0 kb	18197	Hoopes et al., 2018
17	*Camellia sinensis*	I	eudi	3000	3020	80.9	20.0 kb	449.5 kb	36951	Xia et al., 2017
18	*Camptotheca acuminata*	I	eudi	503	403	35.6	108 kb	1752 kb	31825	Zhao et al., 2017
19–1	*Cannabis sativa*	I, R	eudi	820	534	NA	NA	16.2 kb	30000	vanBakel et al., 2011
19–2	*Cannabis sativa*	I, P	eudi	843	808	74.8	513.6 kb	83 Mb	38828	Gao et al., 2020
20–1	*Capsicum annuum*	I	eudi	3260	3349	80.9	55.4 kb	1226.8 kb	35336	Qin et al., 2014
20–2	*Capsicum annuum*	I	eudi	3480	3060	76.4	30 kb	2.47 Mb	34903	Kim S. et al., 2014
21	*Carthamus tinctorius*	I, P	eudi	1170	1060	60.1	21.23 Mb	88.21 Mb	33343	Wu et al., 2021
22	*Catharanthus roseus*	I	eudi	738	523	NA	NA	26.2 kb	33829	Kellner et al., 2015
23	*Centella asiatica*	I	eudi	430	430	56.4	NA	15.7 Mb	25226	Pootakham et al., 2021a
24	*Cerasus humilis*	I, P	eudi	228	223	43.1	1.45 Mb	26.23 Mb	26821	Wang et al., 2020
25	*Chimonanthus praecox*	I, P	magno	779	695	47.5	2.19 Mb	65.35 Mb	23591	Shang et al., 2020
26	*Chimonanthus salicifolius*	I, P	magno	836	820	57.7	2.3 Mb	NA	36651	Lv Q. et al., 2020
27	*Chiococca alba*	I	eudi	567	558	NA	NA	2.35 Mb	28707	Lau et al., 2020
28	*Chrysanthemum nankingense*	I, O	eudi	3240	2530	69.6	130.7 kb	NA	56870	Song et al., 2018
29	*Cinnamomum kanehirae*	I, P	magno	824	731	48.0	0.9 Mb	50.4 Mb	27899	Chaw et al., 2019
30	*Citrus medica*	I	eudi	407	405	43.8	46.5 kb	367 kb	32579	Wang et al., 2017
31	*Citrus reticulata*	I	eudi	334	334	50.1	24.7 kb	1.7 Mb	28820	Wang L. et al., 2018
32	*Coix aquatica*	I, P	mono	1680	1620	75.4	2.24 Mb	NA	39629	Guo et al., 2020
33–1	*Coix lacryma-jobi*	I, P	mono	1800	1730	77.7	3.19 Mb	13.98 Mb	44485	Liu H. et al., 2020
33–2	*Coix lacryma-jobi*	I, P	mono	1560	1280	77.0	NA	594.3 kb	39574	Kang et al., 2020b
34	*Colocasia esculenta*	I, P, O	mono	2390	2405	88.4	400 kb	159.4 Mb	28695	Yin et al., 2021
35–1	*Coptis chinensis*	I, P	eudi	1047	958	62.2	1.58 Mb	4.53 Mb	34109	Chen D. et al., 2021
35–2	*Coptis chinensis*	I, O	eudi	1150	937	62.5	806.6 kb	NA	41004	Liu et al., 2021
36	*Coriandrum sativum*	I, P	eudi	2130	2119	80.6	604.1 kb	160.99 Mb	40747	Song X. et al., 2020
37	*Cuscuta australis*	P, I	eudi	273	265	58.0	3.63 Mb	5.95 Mb	19671	Sun et al., 2018
38	*Dalbergia odorifera*	I, P	eudi	653	638	54.2	5.92 Mb	56.16 Mb	30310	Hong et al., 2020
39	*Datura stramonium*	I, O	eudi	2000	2100	61.0	13.1 kb	164.1 kb	52149	Rajewski et al., 2021
40	*Daucus carota*	I	eudi	473	422	46.0	31.2 kb	12.7 Mb	32113	Iorizzo et al., 2016
41	*Dendrobium catenatum*	I	eudi	1110	1010	78.1	33.1 kb	391 kb	28910	Zhang G. Q. et al., 2016
42–1	*Dendrobium officinale*	I, P	mono	1270	1350	63.3	25.1 kb	76.4 kb	35567	Yan et al., 2015
42–2	*Dendrobium officinale*	I, P	mono	1210	1230	64.4	1.44 Mb	63.07 Mb	27631	Niu et al., 2021
43	*Dimocarpus longan*	I	eudi	445	472	52.9	26.0 kb	566.6 kb	31007	Lin et al., 2017
44	*Dioscorea zingiberensis*	I	mono	851	800	42.8	1.08 kb	1.96 kb	27057	Zhou et al., 2018
45	*Dracaena cambodiana*	I	mono	1120	1064	53.5	1.87 kb	3.19 kb	53700	Ding et al., 2018
46	*Eleutherococcus senticosus*	I, P	eudi	1260	1300	73.6	309.4 kb	50.79 Mb	36372	Yang et al., 2021a
47–1	*Erigeron breviscapus*	I, P	eudi	1520	1200	54.6	18.8 kb	31.5 kb	37504	Yang et al., 2017
47–2	*Erigeron breviscapus*	I, P	eudi	1520	1430	67.4	140.95 kb	156.82 Mb	43514	He et al., 2021
48	*Eriobotrya japonica*	I, P	eudi	803	761	85.9	3.98 Mb	43.16 Mb	43996	Su et al., 2021
49–1	*Eucommia ulmoides*	I, P	eudi	1100	1180	61.2	17.06 kb	1.03 Mb	26723	Wuyun et al., 2018
49–2	*Eucommia ulmoides*	P	eudi	1020	948	62.5	13.16 Mb	53.15 Mb	26001	Li Y. et al., 2020
50	*Fagopyrum tataricum*	I, P	eudi	489	489	51.0	550.7 kb	NA	33366	Zhang L. et al., 2017
51	*Forsythia suspensa*	I, O	eudi	701	737	54.5	7.3 Mb	7.3 Mb	33062	Li L.-F. et al., 2020
52	*Gardenia jasminoides*	I, O	eudi	551	535	62.2	1.0 Mb	44 Mb	35967	Xu et al., 2020b
53–1	*Gastrodia elata*	I	mono	1180	1061	66.2	68.9 kb	4.9 Mb	18969	Yuan Y. et al., 2018
53–2	*Gastrodia elata*	I	mono	1378	1120	69.8	110 kb	1.64 Mb	24484	Chen S. et al., 2020
54	*Gelsemium elegans*	I, O	eudi	338	335	43.2	10.23 Mb	40.47 Mb	26768	Liu Y. et al., 2020
55	*Gelsemium sempervirens*	I	eudi	219	244	NA	NA	411 kb	22617	Franke et al., 2019
56	*Ginkgo biloba*	I	gymno	11750	10610	76.6	48.2 kb	1.36 Mb	41840	Guan et al., 2016
57	*Glycyrrhiza uralensis*	I, P	eudi	401	379	36.5	7.3 kb	109.3 kb	34445	Mochida et al., 2017
58	*Hemerocallis citrina*	I, P	mono	3800	3770	78.9	2.09 Mb	NA	54295	Qing et al., 2021
59	*Hypericum perforatum*	I, P	eudi	400	373	46.9	1.41 Mb	2.31 Mb	29150	Zhou et al., 2021
60	*Isatis indigotica*	I, P	eudi	305	294	53.3	1.18 Mb	36.17 Mb	30323	Kang et al., 2020a
61	*Jacaranda mimosifolia*	I, O	eudi	739	707	56.8	16.77 Mb	39.98 Mb	30507	Wang M. et al., 2021
62–1	*Juglans regia*	I, P	eudi	606	667	51.2	46.1 kb	465.0 kb	32498	Martínez-García et al., 2016
62–2	*Juglans regia*	O	eudi	620	574	58.4	1.1 Mb	37 Mb	37554	Marrano et al., 2020
63	*Lagenaria siceraria*	I	eudi	334	313	46.9	28.3 kb	8.7 Mb	18534	Wu et al., 2017
64	*Lavandula angustifolia*	I, P	eudi	1095	895	58.3	1.22 Mb	36.2 Mb	65905	Li et al., 2021
65	*Lepidium meyenii*	I	eudi	751	743	47.7	81.8 kb	2.4 Mb	96417	Zhang J. et al., 2016
66	*Linum usitatissimum*	I	eudi	373	302	50.0	20.1 kb	693.5 kb	43384	Wang et al., 2012
67	*Lithospermum erythrorhizon*	I, O	eudi	369	367	51.8	314.3 kb	NA	27720	Auber et al., 2020
68	*Litsea cubeba*	I, P	magno	1370	1326	55.5	607.3	1760.0	31329	Chen Y.-C. et al., 2020
69	*Lonicera japonica*	I, O	eudi	887	843	58.2	2.1 Mb	84.4 Mb	33939	Pu et al., 2020
70	*Luffa acutangula*	I, P	eudi	760	735	62.2	NA	786.1 kb	32233	Pootakham et al., 2021b
71–1	*Luffa cylindrica*	I, P	eudi	737	669	62.2	5 Mb	53 Mb	31661	Zhang et al., 2020
71–2	*Luffa cylindrica*	I, P	eudi	720	656	63.8	8.8 Mb	48.76 Mb	25508	Wu H. et al., 2020
71–3	*Luffa cylindrica*	I, P	eudi	773	690	56.8	NA	578.6 kb	43828	Pootakham et al., 2021b
72	*Macleaya cordata*	I	eudi	541	378	43.5	25.0 kb	308.0 kb	22328	Liu et al., 2017
73	*Magnolia biondii*	I, P	magno	2240	2220	66.5	269.1 kb	92.86 Mb	47547	Dong et al., 2021
74–1	*Medicago sativa*/autotetraploid	I, P, O	eudi	3150	2738	55.0	459.0 kb	NA	164632	Chen H. et al., 2020
74–2	*Medicago sativa*Zhongmu No.1 /haploid	I, P	eudi	800	816	57.0	3.92 Mb	NA	49165	Shen et al., 2020
74–3	*Medicago sativa*spp. *caerulea*/diploid	I, O	eudi	802	793	55.6	3.86 Mb	NA	47202	Li A. et al., 2020
75	*Mentha longifolia*	I, P	eudi	400	353	NA	4.5 kb	NA	35597	Vining et al., 2017
76	*Mesua ferrea*	I	eudi	685	614	NA	251.7 kb	392.8 kb	46540	Patil et al., 2021
77	*Mitragyna speciosa*	I	eudi	1123	1123	44.2	70.4 kb	1.02 Mb	55746	Brose et al., 2021
78–1	*Momordica charantia*	I	eudi	339	286	15.3	NA	1.1 Mb	45859	Urasaki et al., 2017
78–2	*Momordica charantia*	I, P	eudi	303	303	52.5	9.9 Mb	25.37 Mb	NA	Matsumura et al., 2020
79	*Morinda officinalis*	I, O	eudi	485	485	58.0	4.2 Mb	40.97 Mb	27102	Wang J. et al., 2021
80	*Moringa oleifera*	I	eudi	278	217	40.6	45.3 kb	957.2 kb	18451	Chang et al., 2019
81	*Morus notabilis*	I	eudi	357	331	47.0	34.5 kb	390.1 kb	29338	He et al., 2013
82	*Myrica rubra*	I	eudi	313	290	45.6	68.6 kb	2164.2 kb	26325	Ren et al., 2019
83–1	*Nelumbo nucifera*	I, R	eudi	929	804	57.0	38.8 kb	3.4 Mb	26685	Ming et al., 2013
83–2	*Nelumbo nucifera*	I	eudi	879	792	49.5	39.3 kb	986.5 kb	36385	Wang et al., 2013
84–1	*Ocimum basilicum*	I	eudi	2360	2068	61.6	48.3 kb	1.5 Mb	78990	Bornowski et al., 2020
84–2	*Ocimum basilicum*	I	eudi	2320	2130	76.0	45.7 kb	19.3 Mb	62067	Gonda et al., 2020
85	*Ocimum tenuiflorum*	I	eudi	612	374	42.9	2.6 kb	27.1 kb	36768	Upadhyay et al., 2015
86	*Ophiorrhiza pumila*	I, P	eudi	440	440	58.2	18.49 Mb	40.06 Mb	32389	Rai et al., 2021
87	*Osmanthus fragrans*	I, P	eudi	741	727	49.4	1.59 Mb	NA	45542	Yang et al., 2018
88	*Paeonia suffruticosa*	I, P	eudi	15760	13790	80.2	49.9 kb	NA	34854	Lv S. et al., 2020
89–1	*Panax ginseng*	I	eudi	3500	3430	62.2	22.0 kb	108.7 kb	42006	Xu et al., 2017
89–2	*Panax ginseng*	I	eudi	3600	2980	79.5	22.5 kb	569.0 kb	59352	Kim et al., 2018
90–1	*Panax notoginseng*	I	eudi	2310	2390	75.9	16.0 kb	96.0 kb	36790	Chen et al., 2017
90–2	*Panax notoginseng*	I	eudi	2002	1850	61.3	13.2 kb	158.0 kb	34369	Zhang D. et al., 2017
90–3	*Panax notoginseng*	O, P	eudi	2310	2240	79.1	220.9 kb	NA	39452	Fan G. et al., 2020
90–4	*Panax notoginseng*	I, P	eudi	2380	2660	85.9	1.12 Mb	216.47 Mb	37606	Jiang et al., 2021
90–5	*Panax notoginseng*	I, P	eudi	2310	2410	88.2	1.45 Mb	196.33 Mb	47870	Yang et al., 2021b
91–1	*Papaver somniferum*	I, O	eudi	2870	2720	70.9	1.77 Mb	2.04 Mb	51213	Guo et al., 2018
91–2	*Papaver somniferum*	I	eudi	3370	2620	65.8	86.0 kb	6.86 Mb	79668	Pei et al., 2021
92–1	*Passiflora edulis*	I, O	eudi	1396	1341	NA	3.1 Mb	NA	23171	Xia et al., 2021
92–2	*Passiflora edulis*	I, P	eudi	1410	1280	86.3	70.2 kb	126.4 Mb	39309	Ma et al., 2021
93	*Phytolacca americana*	I	eudi	1260	930	NA	35.2 kb	42.5 kb	29773	Neller et al., 2019
94	*Piper nigrum*	I, P	magno	762	761	54.9	NA	29.8 Mb	63466	Hu et al., 2019
95	*Platycodon grandiflorus*	I	eudi	683	680	36.2	15 kb	277.1 kb	40017	Kim et al., 2020
96	*Pogostemon cablin* /diploid	I	eudi	1576	1150	58.6	0.4 kb	1.1 kb	45020	He et al., 2016
97	*Pogostemon cablin /*octaploid	I	eudi	2380	1916	43.7	34.7 kb	699.0 kb	110850	He et al., 2018
98	*Polygonum cuspidatum*	I	eudi	2600	2560	71.5	2.8 kb	3.2 kb	55075	Zhang Y. et al., 2019
99	*Poncirus trifoliata*	I, P	eudi	265	265	42.6	842.8 kb	27.7 Mb	25538	Peng et al., 2020
100–1	*Punica granatum*	I	eudi	357	328	46.1	67.0 kb	1.89 Mb	29229	Qin et al., 2017
100–2	*Punica granatum*	I	eudi	336	274	51.2	97.0 kb	1.7 Mb	30903	Yuan Z. et al., 2018
100–3	*Punica granatum*	I, P	eudi	313	320	50.9	4.49 Mb	39.96 Mb	33594	Luo et al., 2020
101	*Raphanus sativus*	I	eudi	529	402	26.7	NA	46.3 kb	61572	Kitashiba et al., 2014
102	*Rhodiola crenulata*	I	eudi	420	345	66.2	25.4 kb	144.7 kb	31517	Fu et al., 2017
103	*Ricinus communis*	Sa	eudi	320	351	50.3	21.1 kb	496.5 kb	31237	Chan et al., 2010
104–1	*Rosa chinensis*	I, P	eudi	560	560	67.9	NA	24 Mb	36377	Raymond et al., 2018
104–2	*Rosa chinensis*	I, P	eudi	568	512	63.2	3.4 Mb	NA	39669	Hibrand Saint-Oyant et al., 2018
105	*Rosa roxburghii*	I	eudi	481	409	47.6	1.5 kb	3.6 kb	22721	Lu et al., 2016
106	*Rosmarinus officinalis*	I	eudi	1180	1014	54.7	21.8 kb	368.7 kb	51389	Bornowski et al., 2020
107	*Salvia bowleyana*	I, P	eudi	462	462	58.7	1.18 Mb	57.96 Mb	44044	Zheng et al., 2021
108–1	*Salvia miltiorrhiza*	I, P, R	eudi	615	538	54.4	12.4 kb	51.0 kb	30478	Xu et al., 2016
108–2	*Salvia miltiorrhiza*	I, P	eudi	572	595	64.8	2.7 Mb	69.8 Mb	32483	Song Z. et al., 2020
109	*Santalum album*	I	eudi	203	221	27.4	460.7 kb	460.7 kb	38119	Mahesh et al., 2018
110–1	*Scutellaria baicalensis*	I, P	eudi	408	387	55.2	880.6 kb	1.34 Mb	28524	Zhao Q. et al., 2019
110–2	*Scutellaria baicalensis*	I, O	eudi	442	377	55.2	2.1 Mb	NA	33414	Xu et al., 2020a
111	*Scutellaria barbata*	I, P	eudi	405	353	53.5	2.5 Mb	NA	41697	Xu et al., 2020a
112	*Selaginella tamariscina*	I, P	lycopo	301	301	60.6	201.2 kb	407.7 kb	27761	Xu et al., 2018
113	*Senna tora*	I, P	eudi	547	526	53.9	4.03 Mb	41.7 Mb	45268	Kang et al., 2020c
114–1	*Sesamum indicum*	I	eudi	357	274	28.5	52.2 kb	2.1 Mb	27148	Wang et al., 2014
114–2	*Sesamum indicum*	P	eudi	337	292	NA	1.06 Mb	20.5 Mb	28406	Li C. et al., 2020
115	*Sinapis alba*	I	eudi	553	459	NA	1.7 kb	NA	34012	Kumari et al., 2020
116–1	*Siraitia grosvenorii*	I	eudi	420	470	NA	34.2 kb	101.1 kb	43856	Itkin et al., 2016
116–2	*Siraitia grosvenorii*	I, P	eudi	420	470	51.1	432.4 kb	NA	30565	Xia et al., 2018
117	*Spatholobus suberectus*	I, P	eudi	793	798	47.8	2.1 Mb	86.99 Mb	31634	Qin et al., 2019
118	*Stevia rebaudiana*	I, O	eudi	1160	1416	80.1	616.9 kb	106.55 Mb	44143	Xu et al., 2021
119	*Taxus wallichiana*	I, O	gymno	10600	10900	85.0	8.6 Mb	987 Mb	44008	Cheng et al., 2021
120	*Toona sinensis*	I, O	eudi	559	596	64.6	1.5 Mb	21.5 Mb	34345	Ji et al., 2021
121	*Trichopus zeylanicus*	I, P	mono	860	714	47.4	289.5 kb	430.0 kb	34452	Chellappan et al., 2019
122	*Trichosanthes anguina*	I, O	eudi	1030	920	80.0	20.11 Mb	82.12 Mb	22874	Ma L. et al., 2020
123	*Tripterygium wilfordii*	P	eudi	366	348	52.4	4.36 Mb	13.52 Mb	28321	Tu et al., 2020
124–1	*Vernicia fordii*	I	eudi	1200	1176	58.7	NA	474.9 kb	46829	Cui et al., 2018
124–2	*Vernicia fordii*	I, P	eudi	1310	1120	73.3	NA	87.15 Mb	28422	Zhang L. et al., 2019
125	*Xanthoceras sorbifolium*	I, P	eudi	442	440	56.4	642.3 kb	29.43 Mb	21059	Liang et al., 2019
126	*Ziziphus jujuba*	I	eudi	444	438	46.8	34.0 kb	301.0 kb	32808	Liu M. J. et al., 2014

*#, number; Esti-size, estimated genome size; Assem-size, assembled genome size; mono, monocots; eudi, eudicots; magno, magnoliids; lycopo, lycopodiophyta; gymno, gymnosperm; Sa, Sanger; R, Roche/454; I, Illumina; P, PacBio; O, Oxford Nanopore; NA, not reported.*

### Research, Protection, and Utilization of Geoherbal Resources

With the widespread application of NGS technology, genome sequencing of medicinal plants has become more feasible due to the greatly reduced cost and time required to complete the project. According to the whole genome sequence, the basic information of biology and biomedical functions can be well understood.

We have made statistics on the medicinal plant genome articles over the years, and have a basic understanding of the general characteristics of the reported medicinal plant genomes. The comparison of size and repetitiveness ratio of these published medicinal plant genomes and their evolution relationship is shown in Figure 2B. Among them, the genomes of five medicinal plants are much larger than other medicinal plants, they are *Allium sativum*, *Paeonia suffruticosa*, *Aloe vera*, *Taxus wallichiana*, and *Ginkgo biloba*. In the plants whose genomes have been sequenced, there are 123 angiosperms (including 12 monocots, 105 eudicots, and 6 magnoliids), two gymnosperms, and one lycopodiophyta plant. The simplified phylogeny of the major clades of sequenced medicinal plants is also shown in Figure 2B. Angiosperms account for the vast majority of sequenced medicinal plants, and eudicots make up the majority of angiosperms. Genome size has a positive correlation with the ratio of repetitive elements, when the genome size is larger, the proportion of repetitive elements also tends to be correspondingly larger. Most of the genome size is concentrated within 4 Gb, and the repetitiveness ratio sequences are concentrated between 30 and 90%.

It has been said that plant genome reports were formulaic and lack biology significance, their descriptions mainly included the assembly, protein-coding genes, repeats, evolution analysis, some aspects of biology, usually with a focus on transcription factors and active compounds biosynthesis pathway (Michael and Jackson, 2013). According to these published medicinal plant genomes, most of them have not yet been used to solve specific application problems, such as discovering new medicinal mechanisms, cultivating new resistant varieties, explaining evolutionary events, and so on. But the assembly of the genomes provides us with the guarantee of the database. Once we need the support of genetic information, the genome is the solid foundation and reference.

## Implications and Hallmark of Medicinal Plant Genome

Medicinal plants are the main sources of medicine, and their records for medicinal usage can be traced back to almost 5,000 years ago in China, India, and Egypt (Moss and Yuan, 2006; Jamshidi-Kia et al., 2018). They are also the precious resource libraries for many chemical drugs, currently, more than one-third of clinical medications are derived from plant extracts or their derivatives (Chen and Song, 2016). The sequencing and demystification of the genome can give us a better understanding of the biosynthesis and regulation of bioactive compounds. Artemisinin-derived plant named *Artemisia annua* is one of the most famous medicinal plants, while the discovery of artemisinin has won the 2015 Nobel Prize in Physiology or Medicine (Su and Miller, 2015). A semi-synthetic system has been used to improve the production of artemisinin greatly (Paddon et al., 2013). Further revealing the genome of *A. annua* provides a comprehensive understanding of artemisinin biosynthesis and leads to improvement in artemisinin production. Before *A. annua* genome revelation, studies manipulating artemisinin biosynthesis focused on either upstream (Nafis et al., 2011) or downstream (Yuan et al., 2015) genes on the artemisinin biosynthesis pathway. Then the combined study and analysis of *A. annua* genomic and associated transcriptomic data proposed other efficient strategies to increase the production of artemisinin, one was to simultaneously enhance the expression of enzyme genes in different steps in the biosynthesis pathway including the upstream (*HMGR*), midstream (*FPS*) and downstream (*DBR2*), and the other was to overexpress the expression of transcription factors like AaMYB2 that could regulate the expression of *ADS*, *CYP71AV1*, *DBR2*, and *ALDH1* in artemisinin biosynthesis pathway, which could significantly improve artemisinin and dihydroartemisinic acid content, providing a new insight for increasing the supply of artemisinin from plant sources (Shen et al., 2018).

In addition to improving the content of active compounds, it is also necessary to ensure the agronomic traits and enhance the resistance ability to stresses of medicinal plants. Genome sequencing can help identify the genes associated with agronomic and disease resistance traits, and can target control of the genes to cultivate new varieties of medicinal plants with highly effective ingredients, excellent agronomic features, and high resistance abilities. *P. notoginseng*, a well-known medicinal plant, is susceptible to a wide range of pathogens, so its cultivation faces several challenges (Ou et al., 2011). The sequencing of the *P. notoginseng* chromosome level genome, combining a genome-wide association study on 240 cultivated individuals, successfully identified 63 genes associated with dry root weight (included genes encoding cysteine/histidine-rich C1 domain proteins), 168 genes associated with stem thickness (included *APC6*, *WRKY71*, and RWA3, etc.) and 33 genes associated with disease resistance trait (included genes encoding LRR receptor-like serine/threonine-protein kinases) (Fan G. et al., 2020). These valuable resources of *P. notoginseng* can provide new opportunities to harness the full potential of its economic and medicinal values.

Moreover, some medicinal plants also play an important role in evolution, and the discovery of their genomes can help to understand the evolutionary relationship of plants. *Ginkgo biloba* is a living fossil without living relatives, which represents one of the four extant gymnosperm lineages (cycads, ginkgo, conifers, and gnetophytes). Its genome showed that LTR-RT insertions and two whole-genome duplications (WGD) events in evolution history contribute to the large genome size and long introns. In angiosperms, chromosomal breakages and fusions, as well as uneven gene loss, might occur to prevent a continuous growth in genome size (Schnable et al., 2009), and this mechanism for removing transposable elements (TEs) might lack and lead to enormous genome size in gymnosperms like ginkgo. The outstanding defense ability of ginkgo resulted from the remarkable duplication of resistance genes and enrichment of relevant pathways. The ginkgo genome sheds light on sequencing large plant genomes and helps to know the genetic and evolutionary process of land plants in natural evolution (Guan et al., 2016).

## Quality and Integrity Improvement of Medicinal Plant Genomes

**FIGURE 3:**
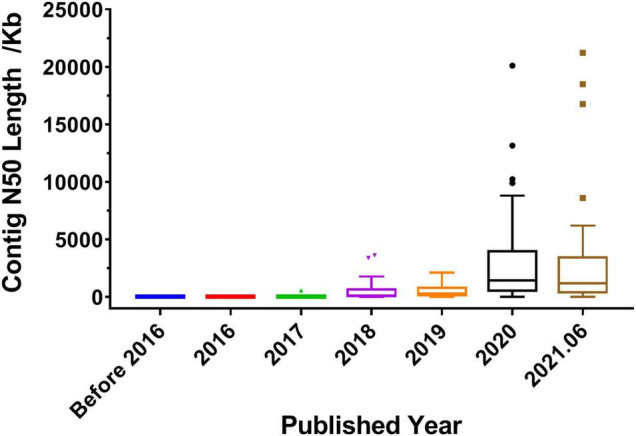
Distribution of contig N50 length in published medicinal plant genomes. Before 2016 represents the published years before 2016, and 2021.06 represents the time between 2021 January and June.

The quality of genome assembly directly affects the quality of the whole genome. Contig N50 and scaffold N50 are the primary indicators for evaluating genome assembly results. Generally, the longer the contig N50 and scaffold N50 are, the better the assembly result is. As shown in Table 1, in 2017 and before, most of the reported medicinal plant genomes used the NGS technologies, such as Illumina and Roche/454, and the length of contig N50 ranged from a few kilobases to dozens of kilobases. In 2018, half of the published genomes used a combination of next- and third-generation sequencing technologies, such as Illumina + PacBio and Illumina + Oxford Nanopore. In 2019 and beyond, the sequencing strategy of combining next- and third-generation has been applied to the majority of the reported genomes. It can be seen from Figure 3 that the length of contig N50 became long since 2018, and then increased year by year. By 2020, the length has been greatly improved, the length of contig N50 was generally increased to the range between a few hundred kilobases and several megabases. The length of contig N50 was similar in the medicinal plant genomes published in 2020 and 2021. And the longest length was as long as 21.23 Mb (Cheng et al., 2021). It shows that the popularization and application of third-generation sequencing have brought convenience to scientific research, and at the same time have greatly improved the quality and integrity of the genome.

## Sequencing Strategy Development

**FIGURE 4:**
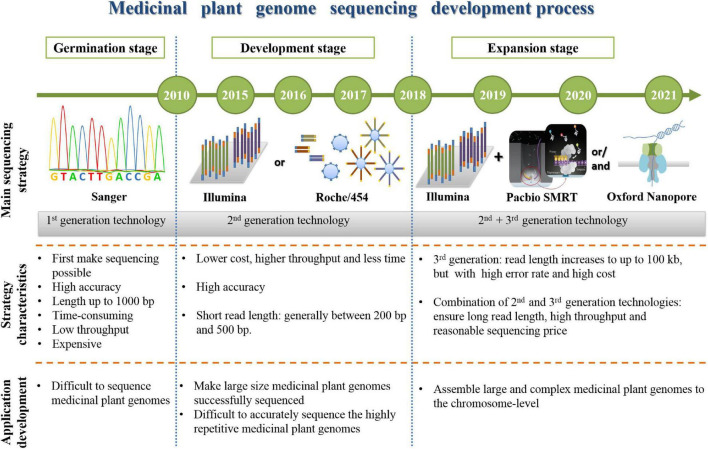
The development process of medicinal plant genome sequencing.

The development process of sequencing strategy on medicinal plant genomes has experienced three stages, germination stage, development stage, and expansion stage (Figure 4).

### Germination Stage of Medicinal Plant Genome Sequencing

The start of genomics is from the early 1990s, and automated sequencing methods using dideoxy chain termination with fluorescent molecules developed, which is known as Sanger sequencing. The effectiveness of the Sanger platform for large eukaryotic genomes was first reported in 2000 for *Drosophila melanogaste*r, ushering in a new era of genomics (Adams et al., 2000). This method was also applied in plant biology, like sequencing ESTs in *Arabidopsis thaliana* (Newman et al., 1994), and then sequencing the whole genome of various plants, like *Oryza sativa* (Yu et al., 2002), *Populus trichocarpa* (Tuskan et al., 2006), *Carica papaya* (Ming et al., 2008) and *Brachypodium distachyon* (International Brachypodium Initiative, 2010). However, there are still gaps and errors in the assembly of these genomes, so they are not completely “finished,” because the process of “finishing” needs inspection and experimental resolution of inconsistencies, and it’s a time-consuming, tough, and expensive work (Hamilton and Robin Buell, 2012). In the germination stage of the development process about medicinal plant genome sequencing, considering this and cost, the Sanger sequencing method is only used to sequence the genome of major economic crops that are also regarded as medicinal plants, like *Ricinus communis*, to provide references and templates for subsequent research.

### The Development Stage of Medicinal Plant Genome Sequencing

After 2011, the NGS technology develops rapidly and occupies the position of the mainstream sequencing platform, becoming the preferred technology for sequencing the medicinal plant genomes. The widely and mainly used NGS platforms are Roche 454 platform and Illumina platform.

Roche 454 platform is the first commercially successful NGS system. This sequencing method uses a high-throughput pyrosequencing technology (Margulies et al., 2005). This platform utilizes emulsion PCR to detect the pyrophosphate released during nucleotide incorporation. In 2005, the read length of Roche 454 was only 100–150 bp with 20 Mb output data per run (Mardis, 2008). In 2008, the 454 GS FLX Titanium system appeared, with a reading length up to 700 bp and 0.7 G output data per run within 24 h. In late 2009, Roche simplified the library preparation and data processing and improved the output to 14 G per run (Liu et al., 2012). In 2012, the platform upgraded to the FLX+ and could generate 1 million reads, with a reading length up to 1,000 bp.

Illumina platform is a high-throughput technology of sequencing by synthesis using reversible dye terminators developed by Solexa and then purchased by Illumina in 2008 (Bentley et al., 2008). The mechanism of the Illumina platform is bridge PCR, which is different from the Roche/454 platform. The library DNA with fixed adaptors is denatured to single strands and linked on the flow cell, followed by bridge amplification to synthesize clusters of clonal DNA fragments. The library splices into single strands by linearization enzyme (Mardis, 2008), and then four kinds of fluorescently labeled nucleotides which have been modified with a terminator complement the template one base at a time, the signal is captured, then the terminator and fluorescent dye are cleaved, and a new round of synthesis repeats until coming up to the desired read length. In late 2011, the paired-end mode of the Hi-Seq2000 Illumina platform could generate more than 250 million reads total sequences of one lane.

Because the throughput of Hi-Seq 2000 is higher, the price is lower, and the application range is wider than Roche/454, the application of the Illumina platform in the medicinal plant genome sequencing occupies the mainstream position. The Illumina platform is widely applied for expression profiling, *de novo* sequencing, and re-sequencing in plant sequencing, like *Thellungiella parvula* (Dassanayake et al., 2011) and *Arabidopsis thaliana* (Cao et al., 2011). As more and more medicinal plant genomes have been reported, the medicinal plant genome sequencing has begun to enter the development stage, many large size medicinal plant genomes were successfully sequenced. However, another difficulty of plant genomes is the high repetition in the genome, so it is difficult to accurately assemble them by the NGS technologies.

### Expansion Stage of Medicinal Plant Genome Sequencing

The development of third-generation sequencing has overcome this problem. The most widely applied long-read sequencing platform is Single-Molecule Real-Time (SMRT) sequencing of Pacific Biosciences company. SMRT sequencing is run on cells, which have tiny wells called zero-mode waveguides (ZMWs). In each ZMWs, a DNA polymerase/template complex gets immobilized, and synthesizes a new DNA strand (Jiao and Schneeberger, 2017). Each incorporation generates a light pulse that can be recognized for differently labeled nucleotides (Eid et al., 2009). PacBio systems can sequence reads with an average size of about 20 kb and a maximum length of over 60 kb (Kim K. E. et al., 2014; Vanburen et al., 2015). Although the sequencing error rate of raw reads is up to 15%, self-correction by adequate coverage sequencing data (Chin et al., 2013) or correction with NGS data (Bashir et al., 2012; Koren et al., 2012) enables genome assemblies with the accuracy of over 99.999% simply by running bioinformatics analysis software (Chin et al., 2016). Besides the PacBio SMRT platform, there is also another long-read sequencing platform introduced by ONT Technologies, which provided access to their first sequencing system in 2014 (Quick et al., 2014; Deamer et al., 2016). Single DNA molecules are run through nanopores, and individual nucleotides create characteristic disruptions in them, which reveal the sequence of the nucleotides. The reads length and sequencing accuracy are similar with PacBio reads, and the longest reads can reach up to 200 kb. First, whole-genome assemblies using ONT data have reached N50 values of multiple hundred kb for fungal genomes, and bacterial genomes could be fully assembled with a nucleotide accuracy of over 99% (Goodwin et al., 2015; Loman et al., 2015).

The emergence of third-generation sequencing technology has made a great leap in sequencing read length and brought medicinal plant genome sequencing into a stage of rapid development. The strategy used in this stage is a combination of second- and third-generation sequencing technologies, which can ensure long read length, high throughput, and reasonable sequencing price at the same time. Medicinal plant genomes are large and have high-ratio repetitive elements, the frequently-used strategy is combining high coverage Illumina and low coverage PacBio SMRT or ONT data. Because third-generation sequencing can provide long-read sequences to increase the assembly accuracy and genome draft quality, but the price is relatively high, so Illumina platform is used to guarantee enough sequencing data. And this can make it possible to assemble large and complex medicinal plant genomes to the chromosome level. After these years of sequencing development, the medicinal plants not only can obtain draft genome relevant information and dig out target protein-coding genes, but also recognize the chromosome-level of the genome to discover the evolution, gene cluster’s function, repetitive elements effect, and so on.

### Genomes of Species Have Been Repeatedly Sequenced

We found that not only does the number of medicinal plant genomes sequenced continue to increase, but the number of medicinal plant genomes sequenced repeatedly is also increasing. Why? First of all, because the genomes of many medicinal plants have not been revealed yet, many teams are performing *de novo* sequencing of the genomes at the same time, and accordingly publish them at the same time. Then, with the continuous development of gene sequencing technology, we can obtain longer sequencing read lengths, so as to assemble more complete and accurate high-level genomes. Genomes assembled to the chromosome level are the current trend. The information that the genome gives us is no longer a contig or scaffold, but the chromosome and the position of a gene on the chromosome.

There are 25 medicinal plants with two reported genomes, three medicinal plants with three reported genomes, and one plant with five reported genomes. Representative medicinal plants include *Momordica charantia* (bitter gourd), *Salvia miltiorrhiza* (Danshen), *Punica granatum* (pomegranate), *Panax notoginseng* (Sanqi), *Panax ginseng* (Asian ginseng), *etc*. Bitter gourd and danshen have two reported versions of the genome. Bitter gourd completed the *de novo* assembly of the genome draft in 2017, as well as basic annotation and evolutionary analysis (Urasaki et al., 2017). In 2020, using PacBio long-read sequencing technology, the *Momordica charantia* genome was assembled to the chromosome level, and further investigate the genomic changes under domestication (Matsumura et al., 2020). The genome of *Salvia miltiorrhiza* was also assembled to eight chromosomes, the assembled genome size increased from 538 to 594.75 Mb, and the proportion of repetitive elements also increased from 54.44 to 64.84% (Xu et al., 2016; Song Z. et al., 2020). *Punica granatum* (pomegranate), which is a popular and nutritious fruit with medicinal properties, has three published genome versions (Qin et al., 2017; Yuan Z. et al., 2018; Luo et al., 2020). The third version of the genome is assembled to the chromosome level, and it is a high-quality genome map of the soft-seed pomegranate, which helps to clarify the genetic divergence between soft- and hard-seeded varieties and provides insights into the genetic diversity and population structure of pomegranates (Luo et al., 2020). *Panax notoginseng* (Sanqi) is a well-known TCM whose genome research is sought after by scientists, and a total of five versions have been reported. The three recent versions are assembled to the chromosome level (Fan G. et al., 2020; Jiang et al., 2021; Yang et al., 2021b), which are more complete than the previously available genome assemblies (Chen et al., 2017; Zhang D. et al., 2017), further reveal the biosynthesis pathways of ginsenosides and dencichine, as well as provide a resource for further exploration of the saponin biosynthesis, cultivation, and breeding of *P. notoginseng*. *Panax ginseng* (Asian ginseng), reputed as the king of medicinal herbs, belongs to the same genus *Panax*, which also has two versions of reported genomes (Xu et al., 2017; Kim et al., 2018). Both of these two genomes provide a comprehensive understanding for functional and evolutionary analysis as well as ginsenoside biosynthesis. Additionally, Kim et al. (2018) identified fatty acid desaturases that can increase freezing tolerance and chlorophyll a/b binding protein genes which enable efficient photosynthesis under low light. However, the read length of both genomes is not long enough by the current standards, and there is still space for further improvement in the integrity and accuracy of the ginseng genome.

## Application of Medicinal Plant Genomes

### Genomics-Assisted Herb Breeding

The genes related to medicinal plant growth and development, disease resistance, important genetic traits, and germplasm characters which are the important functional genes in medicinal plants, taking advantage of genome annotation information, discovering good genes, using genetic engineering methods to break the reproductive isolation, and cultivating the new species with excellent agronomic characters and high content of active ingredients, so that it can lay the foundation for the large-amount extraction of active ingredients and extensive clinical application. By combing transcriptome and resequencing of individual species within or between species, the large-scale molecular markers can be identified rapidly and accurately, and genetic linkage study of molecular markers and qualified characters can also be accelerated, the phenotypes of medicinal plants and the relationship of physical characteristics and genotypes are discovered quickly so that efficiency of breeding are improved obviously.

The study of *Scutellaria baicalensis* (Huangqin) genome sequencing revealed that a specialized metabolic pathway for the synthesis of 40-deoxyflavonebioactives evolved in the genus *Scutellaria* and found that the gene encoding a specific cinnamate coenzyme A ligase likely obtained its new function following recent mutations and that four genes encoding enzymes in the 40-deoxyflavone pathway are present as tandem repeats in the genome of Huangqin. Further analysis discovered that gene duplications, segmental duplication, gene amplification, and point mutations coupled to gene neo- and subfunctionalizations were involved in the evolution of 40-deoxyflavone synthesis in *Scutellaria*. These results not only provide significant insight into the evolution of specific flavone biosynthetic pathways in the mint family *Lamiaceae* but also facilitate the development of tools for enhancing bioactive productivity by molecular breeding in plants (Zhao Q. et al., 2019).

### Evolution History Revealing

**FIGURE 5:**
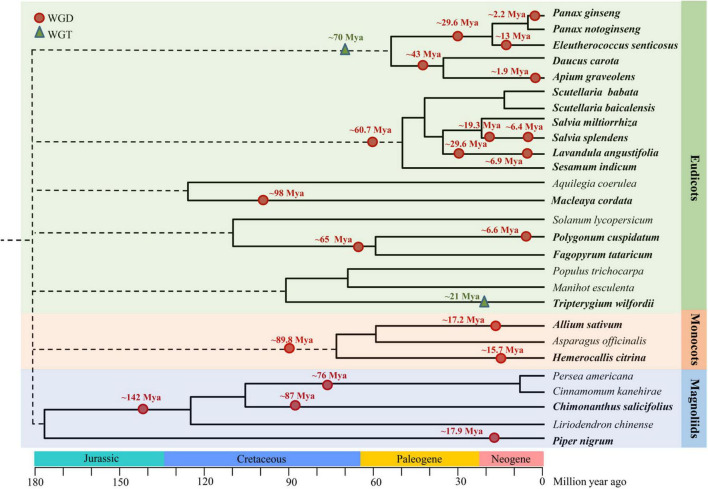
The whole genome duplication (WGD) and whole genome triplication (WGT) events in representative medicinal plants.

Whole-genome sequencing cannot only elucidate the biosynthesis pathways of natural products but also give insight into their evolution. The evolution will bring the whole genome change, like WGD and whole-genome triplication (WGT), to adapt to the environment alteration and explain the characters of plants. We summarized the WGD and WGT events of some representative species reported in the medicinal plant genome articles, and these situations are shown in Figure 5. These WGD and WGT events are summarized and introduced into three types of plants, which are eudicots, monocots, and magnoliids.

In the eudicots part, we select five representative branches to demonstrate the situation. The representative medicinal plants of Araliaceae and Apiaceae are clustered together. *P. ginseng*, *P. notoginseng*, and *E. senticosus* belong to Araliaceae, and *P. notoginseng* is diploid, while *P. g*inseng and *E. senticosus* are tetraploid. Two rounds of WGD were discovered in these Araliaceae plants, the first round occurred around 29.6 Mya, *P. ginseng*, and *E. senticosus* both had the second round of WGD, which were found almost 2.2 Mya in *P. ginseng* and 13 Mya in *E. senticosus*, respectively. Additionally, these recent WGDs were discovered to contribute to the ability of *P. ginseng* to overwinter and *E. senticosus* to adapt to cold environment, enabling them to live and spread broadly through the cold area (Kim et al., 2018; Jiang et al., 2021; Yang et al., 2021a). These two rounds of WGD occurred in the family Araliaceae after divergence with the Apiaceae, which may be one of the reasons why its genome was bigger than other medicinal plants. In the *D. carota* and *A. graveolens* that belonged to Apiaceae, one shared WGD occurred in about 43 Mya, and one recent WGD only existed in *A. graveolens* in approximately 1.9 Mya, and this duplication contributed to the expansion of terpene synthase gene families (Song X. et al., 2020). The second branch in the eudicots part includes six plants belonging to Lamiales, one shared WGD (almost 60.7 Mya) was identified in *S. baicalensis*, *S. barbata*, *S. miltiorrhiza*, and *S. indicum*, which might be responsible for chromosomal expansion and rearrangement (Xu et al., 2020a), and two rounds of WGD were found in *S. splendens* and *L. angustifolia*, which could result in the gene families expansion related to terpenoid biosynthesis (Li et al., 2021). In *P. cuspidatum*, it experienced current lineage-specific WGD at 6.6 Mya after the divergence with *F. tataricum* from the ancestor, and it shared the ancient and common WGD with *F. tataricum* at 65 Mya (Zhang Y. et al., 2019), after this WGD, the genome of *F. tataricum* experienced dramatic chromosomal rearrangements, resulting in very fragmented intra-genome collinear blocks (Zhang L. et al., 2017). There is also a WGT event identified and reported in the medicinal plant genome articles. *T. wilfordii* was found to have a WGT event in approximately 21 Mya, which enabled it to cope better with and adapt to the markedly changed environment, and the duplication of the triptolide biosynthesis genes were almost generated by this WGT event, suggesting this WGT event was important to the evolution of triptolide biosynthesis (Tu et al., 2020).

In the monocots part, *A. sativum* and *H. citrina* are the representatives. *A. sativum* has undergone two rounds of WGD, suggesting WGD can be the important driving force of the proliferation of TEs and genome expansion in garlic (Sun et al., 2020). Otherwise, *H. citrina* experienced a recent WGD event at about 15.73 Mya, which was the main factor resulting in multiple copies of the orthologous genes (Qing et al., 2021). In the magnoliids part, *C. salicifolius* and *P. nigrum* are the representatives. Two rounds of ancient WGD were inferred in the *C. salicifolious* genome, one was shared by Calycanthaceae at ∼87 Mya after its divergence with Lauraceae, and the other was dating back to approximately 142 Mya in the ancestry of Magnoliales and Laurales (Lv Q. et al., 2020). Meanwhile, the *P. nigrum* genome was speculated to have a WGD event at ∼17.9 Mya, which brought genetic changes that were responsible for the particular biosynthesis of piperine (Hu et al., 2019).

### Domestication Process Understanding

Domestication is a complex evolutionary process, which is one of the most important technological innovations in human history, humans use plants to change their morphology and physiology traits, distinguishing them from wild ancestors, and ultimately giving rise to the current human cultures (Diamond, 2002; Hancock, 2005). Some of the domesticated plants are medicinal plants. The timing and geographical origins of domesticated traits, as well as the genes that lead to changes in traits, can be sent to find clues from genomic information (Purugganan and Fuller, 2009).

Coix is a widely cultivated grass crop with high nutritional and medicinal value, which has been domesticated as early as the Neolithic era. However, its genetic research and breeding were hampered by the lack of a sequenced genome. Two chromosome-level genomes of coix have been reported simultaneously, which belong to elite cultivar Beijing (Liu H. et al., 2020) and wild relative *Coix aquatica* Daheishan (Guo et al., 2020), respectively. They both find that hull thickness is an important domestication trait between the wild relatives and cultivars, and selection of papery hull from the stony hull in wild progenitors was a key step in coix domestication. Combining resequencing analysis and comparative analysis, several domesticated loci or genes (like loci in the ∼2 to 150 kb region upstream of *ub3*) and two major quantitative trait loci associated with hull thickness and color (Ccph1 and Ccph2), were discovered to be the potential identification loci for domestication. These findings will greatly facilitate and benefit the molecular breeding of coix and provide a powerful reference for the domestication and evolution of medicinal plants.

### Herbal Synthetic Biology

The active components of medicinal plants with complex and diverse structures are the material basis for their medicinal effect, and it’s also an important source of new drug discovery. However, many medicinal plant materials often face a series of problems in the process of development and utilization, for example, the growth of many medicinal materials is greatly affected by environmental factors; some rare herbs grow slowly and are difficult to grow by artificial cultivation; most of the active ingredients are low in content, complex in chemical structure and difficult in chemical synthesis; traditional methods of natural extraction or artificial chemical synthesis cannot meet the needs of scientific research and new drug development. Synthetic biology will be an effective way to resolve these problems.

As high-throughput sequencing technology for genome and transcriptome studies have developed rapidly, using bioinformatics method and functional genomics approach to screen and identify enzyme-coding genes on specific secondary biosynthesis pathway from a large number of the original species of medicinal plants, which will greatly accelerate the analysis process of secondary biosynthesis pathway and lay a solid foundation for herbal synthetic biology research of medicinal plants.

*Tripterygium wilfordii* genome is one of the typical examples. Because of the extremely low yield of triptolide extracted from *T. wilfordii*, its original plant cannot be grown on a large scale, and the current chemical synthesis route is limited to a yield of less than 1.64%. A more promising method to obtain more triptolide could be metabolic engineering, which can be realized via a synthetic biology strategy. However, it required elucidation of the triptolide biosynthesis pathway. Therefore, the sequencing of the *T. wilfordii* genome was completed, and cytochrome P450 TwCYP728B70 involved in triptolide biosynthesis was identified, accordingly, the triptolide content in the CYP728B70 overexpression line increased obviously (Tu et al., 2020). It’s important to make full use of genomic resources to reveal the biosynthesis pathways of active compounds in medicinal plants and use candidate genes in these pathways for the heterologous bioproduction under synthetic biology strategy.

### Geoherbal Research, Protection, and Utilization of Resources

Geoherbs, controlled by genetic factors and affected by environmental conditions, are representative of high-quality medicinal materials. The utilization of sequencing technology and data can provide useful tools to elucidate the molecular mechanism of geoherbs. For the same medicinal plants in different areas, epigenomic studies of medicinal plants can be carried out to clarify the genetic variation of different production areas, especially the modification effect of different environments on the epigenome of medicinal material, including DNA methylation modification, small RNA sequencing analysis, chromatin immunoprecipitation analysis, and so on. In addition, microorganisms in soil are also important factors in the growth environment of geoherbs. Metagenomic analysis of soil microbial community can be sequenced to provide the basis for revealing the interaction between soil microorganisms and the growth of medicinal plants.

Recently, 545 genomes of ginkgo trees sampled from 51 populations across the world were sequenced to identify three refugia in China and detect multiple cycles of population expansion and reduction along with glacial admixture between relict populations in the southwestern and southern refugia, and multiple anthropogenic introductions of ginkgo were proved to occur from eastern China into different continents. This study provides insight into the evolutionary history of ginkgo and helps to provide protection and utilization way for its valuable genomic resources (Zhao Y. P. et al., 2019).

### Improving the Synthesis Efficiency of Bioactive Compounds Within Species

Because of the rapid development and progress of sequencing technology, more and more biosynthesis pathways of active ingredients from medicinal plants have been revealed. The early-stage was based on the mining from transcriptome data, and the later stage was based on the combined mining from genome and transcriptome data. Although transcriptome sequencing has so far occupied a major position in the research of biosynthesis pathways of medicinal ingredients, genome data can provide more important information, for example, it can reveal the evolution process of biosynthesis pathway genes, thereby efficiently synthesizing secondary metabolites with medicinal activity. In the opium poppy genome, a great discovery about a gene cluster including 15 genes was reported. Meanwhile, in its evolution process, the events like gene duplication, rearrangement, and fusion, could lead to the aggregation and co-expression of genes in the two metabolic pathways of noscapine and morphinan, so that it resulted in the formation of this supergene cluster, which could synergistically synthesize the medicinal ingredients in opium poppy (Guo et al., 2018). Therefore, the opium poppy genome helps to decipher the mystery of the synthesis of secondary metabolites. It is not only beneficial to the development of molecular plant breeding tools and cultivating new varieties, but also has great guiding significance for the selective improvement of the production of alkaloids with different efficacy in future artificial synthesis.

It also provides new ideas for the application of medicinal plant genomes. Through the evolution process, gene duplications and neofunctionalization can generate gene clusters, which may relate to specialized metabolites, and this phenomenon has already been observed in several model plants, like *A. thaliana*, *Zea mays*, and *Solanum lycopersicum* (Bharadwaj et al., 2021). In medicinal plants, we can refer to the research strategy of the opium poppy (Guo et al., 2018), which can help us understand the formation process of gene clusters related to medicinal active ingredients and improve their biosynthesis efficiency.

### Comparative Genomic Analysis Among Different Species or Different Populations in the Same Species

The continuous emergence of high-quality genomes has made the application of comparative genomics analysis more and more extensive and in-depth, and it is also a powerful tool for researchers to dig out biological problems and explain biological phenomena (Nobrega and Pennacchio, 2004). Comparative genomics, based on genome mapping and sequencing technology, are generally referred to as comparative analysis of the structural and functional gene regions of the genomes among multiple species or multiple individuals (populations) from one species. Specifically, it is to compare the similarities and differences in the structural characteristics, study the contraction and expansion of gene families, discover the differentiation time and evolution relationship, analyze the generation and evolution of new genes, *etc*.

One representative example of comparative genomics among different medicinal plant genome species can be *Scutellaria baicalensis* and *Scutellaria barbata*. The comparative genomic analysis of them showed the recent LTR may result in chromosomal rearrangement and expansion, and tandem duplication of paralogs after their speciation might contribute to the divergent evolution of flavonoid biosynthesis gene families, which provided a significant foundation for the evolution and chemodiversity studies in the Lamiaceae (Xu et al., 2020a).

Moreover, a representative of comparative genomics among different populations in the same species can be *Forsythia suspense*. Genome-wide comparative analysis was then conducted for the 15 natural populations across its current distribution range. The results revealed that candidate genes associated with local adaptation were functionally correlated with heterogeneous environmental factors, and supported the hypothesis that adaptive differentiation should be highly obvious in the genes of signal crosstalk between different environmental variables, which gave insights into the fundamental genetic mechanisms of the local adaptation to climatic gradients in plant species (Li L.-F. et al., 2020).

## Outlook and Challenges of Medicinal Plant Genome Sequencing

The use of medicinal plants has a long history and diverse application methods. Related works of research mainly focus on the discovery of chemical basis and the analysis of pharmacodynamic effect, but the understanding of medicinal plant genetic resources is relatively weak. Therefore, the research on the genome of medicinal plants should make use of the latest technologies and achievements of genomics, and integrate the studies of structural genome, functional genome, transcriptome, proteome, epigenome, metagenome, synthetic biology, metabolome, bioinformatics, and other relevant databases. Therefore, the essence of medicinal plants can be revealed, the relationship among genetic resources, chemical quality, and drug efficacy can be recognized.

We are most concerned about the medicinal value of medicinal plants. The medicinal value is not only reflected in the content of their medicinal ingredients, but also the stability of the quality of their medicinal materials. Now medicinal plant genomes can be annotated to obtain protein-coding genes, especially biosynthesis genes of active ingredients, analyze their evolutionary history and domestication process, and discover genes that respond to environmental stresses to help improve their resistance and ability. However, the powerful ability of the medicinal plant group has not yet been manifested, and its ability to solve the difficulties in practical applications remains to be developed. How to use the information of the medicinal plant genome to transform and obtain excellent medicinal plant varieties has not yet been realized. Determining suitable model medicinal plants is of great significance to the research on the practical application of medicinal plant genomes. The determination of appropriate model medicinal plants is of great significance to the study of the genomics of medicinal plants. From the perspective of general biological characteristics, it usually should have the traits of a short age cycle, many offspring, and stable phenotype. As for genetic resources, the genome should be relatively small, easy to sequence, and genetic transformation is relatively easy. As for medicinal characteristics, it should be suitable for secondary metabolite biosynthesis and production research. Therefore, the establishment and improvement of a suitable model medicinal plant platform will greatly enhance the application value of medicinal plant genomes.

The assembly of plant genomes is a challenging problem because of their high repetitiveness due to TEs, extreme genome sizes, and polyploid nature. With the development and emergence of long-read sequencing (Eid et al., 2009; Deamer et al., 2016) and long-range scaffolding methods such as optical mapping (Schwartz et al., 1993), chromosome conformation capture (Burton et al., 2013), and DNA dilution-based technologies (Amini et al., 2014; Zheng et al., 2016), the medicinal plant genome sequencing overcomes weaknesses of short-read assemblies and becomes possible to assemble to the chromosome-level (Jiao and Schneeberger, 2017). Although there have been medicinal plants that enable the assembly of entire chromosomes, most medicinal plants just still obtained long scaffolds or super-scaffolds. And now we have got a large amount of sequencing data from medicinal plants, how to effectively explore and apply them to dig deeper information is still facing problems and challenges.

Moreover, thanks to the advancement and development of sequencing technology and bioinformatics algorithms, at least one hundred medicinal plant genomes have been obtained. How to use them thoroughly and effectively has attracted the attention of many institutions and researchers. In recent years, several databases of medicinal plant genomes have already been built, such as the Herbal Medicine Omics Database^1^ (Wang X. et al., 2018), 1K Medicinal Plant Genome Database,^2^ and Database of 10,000 Medicinal Plants.^3^ These databases summarize the medicinal plant genomes that have been reported at this stage or aim to build a biological big data platform for medicinal plants, linking the omics data, active ingredients, disease information, and other information to promote their modernization. All of the above indicate that the medicinal plant genome has entered the stage of big data association research from the stage of exploring the unknown. Moreover, because of the limitations of previous technologies and methods, the disclosed medicinal plant genome information is limited. If the obtained medicinal plant genome information is aggregated and shared through the database, this should be a huge treasure to be unearthed, which will prompt the research efficiency of medicinal plants.

## Conclusion

Thanks to the invention of the long-read sequencing technology, the research on medicinal plant genomes has developed rapidly and is no longer limited by their huge genome size and high repetitive sequences. The number of genomes reported in the past 2 years has increased significantly, and the quality of genomes has also been greatly improved, most of which have been assembled to the chromosome level. Correspondingly, the sequencing strategy they adopted has also been continuously updated, making them more and more widely used, answering and solving many problems in scientific researches and practical applications, including herb breeding assistance, evolutionary history revealing, domestication process understanding, herb synthetic biology study, geoherbal research and comparative genome analysis, these are of great significance to the effective use and sustainable protection of medicinal plants, which can improve their research efficiency and promote their modern development.

## Author Contributions

Q-QC planned the manuscript outline, wrote the draft, and created the figures and tables. YO, Z-YT, C-CL, Y-YZ, and C-SC proofread the manuscript. HZ supervised the study and revised the manuscript. All authors contributed to the article and approved the submitted version.

## Conflict of Interest

The authors declare that the research was conducted in the absence of any commercial or financial relationships that could be construed as a potential conflict of interest.

## Publisher’s Note

All claims expressed in this article are solely those of the authors and do not necessarily represent those of their affiliated organizations, or those of the publisher, the editors and the reviewers. Any product that may be evaluated in this article, or claim that may be made by its manufacturer, is not guaranteed or endorsed by the publisher.

## Footnotes

1
http://herbalplant.ynau.edu.cn/

2
http://www.herbgenome.com

3
https://db.cngb.org/10kmp/
