# Supplementary material for: Review on the Development and Applications of Medicinal Plant Genomes
Source: Front Plant Sci. 2021 Dec 23;12:791219. doi: 10.3389/fpls.2021.791219 (PMC8732986; doi:10.3389/fpls.2021.791219)
Supplement: Supplementary file 1 [file Table_1.docx]

**Table S1｜**The corresponding journal name and article number.

| **No.** | **Journal Name** | **Article Number** |
| --- | --- | --- |
| 1 | Horticulture Research | 25 |
| 2 | Molecular Plant | 21 |
| 3 | The Plant Journal | 13 |
| 4 | Nature Communications | 12 |
| 5 | GigaScience | 11 |
| 6 | Molecular Ecology Resources | 8 |
| 7 | Plant Biotechnology Journal | 7 |
| 8 | DNA Research | 5 |
| 9 | Nature Genetics | 5 |
| 10 | PNAS | 4 |
| 11 | BMC Genomics | 3 |
| 12 | Frontiers in Genetics | 3 |
| 13 | Frontiers in Plant Science | 3 |
| 14 | G3 | 3 |
| 15 | Genomics Proteomics Bioinformatics | 2 |
| 16 | Iscience | 3 |
| 17 | PLoS ONE | 3 |
| 18 | Acta Pharmaceutica Sinica B | 2 |
| 19 | Genome Biology | 2 |
| 20 | Scientific Data | 2 |
| 21 | Scientific Reports | 2 |
| 22 | BMC Biology | 1 |
| 23 | BMC Plant Biology | 1 |
| 24 | ChemBioChem | 1 |
| 25 | Ecology and Evolution | 1 |
| 26 | Gene | 1 |
| 27 | Genome | 1 |
| 28 | Genome Biology and Evolution | 1 |
| 29 | Genomics | 1 |
| 30 | Journal of Integrative Plant Biology | 1 |
| 31 | Journal of Pineal Research | 1 |
| 32 | Nature Biotechnology | 1 |
| 33 | Nature Plants | 1 |
| 34 | New Phytologist | 1 |
| 35 | Plant and Cell Physiology | 1 |
| 36 | Plant Cell Physiology | 1 |
| 37 | Plant Communications | 1 |
| 38 | Plant Genome | 1 |
| 39 | Plant Physiology | 1 |
| 40 | Science | 1 |
